# *Microbial Biotechnology* 2022 and onwards

**DOI:** 10.1111/1751-7915.13969

**Published:** 2021-11-12

**Authors:** Juan L. Ramos

**Affiliations:** ^1^ Consejo Superior de Investigaciones Científicas Estación Experimental del Zaidín 18008‐Granada Spain

It is a real privilege for me to be appointed as the Editor‐in‐Chief of *Microbial Biotechnology (MBT)* – a journal of the Society for Applied Microbiology, the UK’s oldest microbiology society. Since 2008, I served as the Editor of MBT, when it was founded by Kenneth Timmis with the vision of highlighting the ever‐changing interface between biotechnology and microbiology.

Today, the journal is renowned in the field thanks to the efforts of past (Marty Rosemberg, Willy Verstraete, Willem de Vos, Siegfried Vlaeminck, Roland Siezen) and present editors (Harald Brüssow Pablo Nikel, Auxiliadora Prieto, Sepideh Pakpour, Davinia Salvachua, Hui Wang, Antonie Danchin, Carmen Michán, Wei Huang and Larry Wacket), editorial board members and *ad hoc* reviewers. Combined, these devoted and highly skilled individuals have helped to guide strategic direction of the journal, while helping to select and review the world’s top articles for our readers. I look forward to continuing in these endeavours. Some of my earliest efforts have been focused on further enriching the diversity of our editors and to recruit early‐career scientists to our Editorial Board – initiatives that will enable the journal to engage with a wide spectrum perspective (a key requirement for innovation).

During the first 15 years, *MBT* was published bimonthly, but starting in 2022 with this issue, the journal will be published on a monthly basis. Since its beginnings, *MBT* has published both regular and special issues focused on biotechnology – from basic science to applied microbiology and process development. While regular issues serve to highlight research related to any area of microbial biotechnology, special issues feature articles related to specific themes. *MBT* will keep this basic structure with regular articles (minireviews, research articles and brief reports), as well as other articles, such as editorials, opinion articles, Lilliput, web alerts and correspondence. Starting in 2022, a new type of article known as ‘Patent features’ will be added. This article type will provide a venue for industrial scientists to highlight their work, while promoting collaboration between industry and academia.

Every other year, *MBT* publishes visionary papers in the pioneering Crystal Ball Special Issue – these papers highlight the brightest biotech developments across the full spectrum of *in silico* to applied research. Beginning in 2022, we will begin publishing this issue more frequently, on an annual basis, to provide our readers with even more relevant, future‐focused and visionary ideas.

At our readers’ request, *MBT* will intensify its coverage of Engineering Biology and Synthetic Biology. This focus comes on the heels of the launch of the Synthetic Biology Special Issue in December 2021. This focus will promote multidisciplinary research to create novel, unexpected solutions for the development of eco‐friendly production processes.

We will continue to work closely with Wiley – our publishing house – to facilitate and streamline the submission, peer‐review and publication process.

Combined, these efforts will serve to advance the following goals: (i) to ensure that *MBT* continues to serve as a vital link between all the various players in the field; (ii) to place the journal as the leading venue for sharing and discussing the advancements in the field; and (iii) to spark innovation and new industrial applications and jobs in this burgeoning area.

I look forward to working with you and our community – and to doing so in open and inclusive way. Join me in exploring new ideas, developing skills and in widening our understanding of the exciting and dynamic world of microbial biotechnology.

## Conflict of interest

None declared.

## Funding Information

No funding information provided.

